# The basal position of nuclei is one pre-requisite for asymmetric cell divisions in the early mouse embryo

**DOI:** 10.1016/j.ydbio.2014.05.009

**Published:** 2014-08-15

**Authors:** Anna Ajduk, Sourima Biswas Shivhare, Magdalena Zernicka-Goetz

**Affiliations:** a The Wellcome Trust/Cancer Research UK Gurdon Institute, University of Cambridge, Tennis Court Road, Cambridge CB2 1QN, UK; b Department of Physiology, Development and Neuroscience, University of Cambridge, Downing Street, Cambridge CB2 3DY, UK; c Department of Embryology, Faculty of Biology, University of Warsaw, Miecznikowa 1, 02-096 Warsaw, Poland; d Institute of Reproductive Sciences, University of Oxford, Oxford OX4 2HW, UK

**Keywords:** Mammalian embryo, Preimplantation development, Division plane, Cell polarity, Cytoskeleton

## Abstract

The early mouse embryo undertakes two types of cell division: symmetric that gives rise to the trophectoderm and then placenta or asymmetric that gives rise to inner cells that generate the embryo proper. Although cell division orientation is important, the mechanism regulating it has remained unclear. Here, we identify the relationship between the plane of cell division and the position of the nucleus and go towards identifying the mechanism behind it. We first find that as the 8-cell embryo progresses through the cell cycle, the nuclei of most – but not all – cells move from apical to more basal positions, in a microtubule- and kinesin-dependent manner. We then find that all asymmetric divisions happen when nuclei are located basally and, in contrast, all cells, in which nuclei remain apical, divide symmetrically. To understand the potential mechanism behind this, we determine the effects of modulating expression of Cdx2, a transcription factor key for trophectoderm formation and cell polarity. We find that increased expression of Cdx2 leads to an increase in a number of apical nuclei, whereas down-regulation of Cdx2 leads to more nuclei moving basally, which explains a previously identified relationship between Cdx2 and cell division orientation. Finally, we show that down-regulation of aPKC, involved in cell polarity, decreases the number of apical nuclei and doubles the number of asymmetric divisions. These results suggest a model in which the mutual interdependence of Cdx2 and cell polarity affects the cytoskeleton-dependent positioning of nuclei and, in consequence, the plane of cell division in the early mouse embryo.

## Highlights

• In 8-cell embryo most of the nuclei move from apical to basal positions.
• The nuclear movement depends on microtubules and kinesins.
• Only blastomeres with basally located nuclei can divide asymmetrically.
• Blastomeres with apically located nuclei divide only symmetrically.
• Position of the nucleus is regulated by interdependence of Cdx2 and cell polarity

## Introduction

In the mouse embryo, division from the 8 to 16 cell stage leads to segregation of progenitors for two distinct lineages: outer cells that give rise to the extra-embryonic trophectoderm contributing to the placenta, and inner cells that give rise to the inner cell mass (ICM) contributing to the embryo proper. As the blastocyst forms, inner cells are wholly surrounded by neighbouring cells, whereas outer cells contact other cells only on one side. One view, the ‘inside–outside’ hypothesis, suggests that this cell–cell apposition and specific inside microenvironment results in a distinct cell fate (Tarkowski and Wroblewska, 1967; Yamanaka et al., 2006). The alternative model suggests that cell fate is determined by cell polarity that is established along the apical–basal axis (Johnson and Ziomek, 1981b). Cell polarity is manifested by both cell morphology, for example formation of microvilli in the apical domain (Reeve and Ziomek, 1981), and on the molecular level, for example by apical accumulation of aPKC, Par6, Par3 proteins (Plusa et al., 2005; Vinot et al., 2005) and transcripts of Cdx2 (Skamagki et al., 2013). Consequently, a symmetric division (parallel to the apical–basal axis) gives rise to outer, polarized cells and an asymmetric division (orthogonal to the apical–basal axis) produces an outer, polarized cell and an inner, unpolarized cell (Johnson and Ziomek, 1981a; Zernicka-Goetz et al., 2009). Early observations showed that indeed 8–16 cell divisions are either parallel or orthogonal in respect to the apical–basal axis resulting in either two polar or polar and apolar daughter cells, but never in two apolar couplets (Johnson and Ziomek, 1981a; Sutherland et al., 1990). More recently, molecular markers predestining cells to asymmetric or symmetric divisions have been identified. A tendency of cells to undergo symmetric or asymmetric divisions has been correlated with expression of Cdx2, key for trophectoderm formation (Jedrusik et al., 2008, 2010; McDole and Zheng, 2012; Niwa et al., 2005; Ralston and Rossant, 2008; Strumpf et al., 2005). When the level of Cdx2 expression is high, cells tend to divide symmetrically and give rise to trophectoderm, while, in contrast, Cdx2 depletion leads to preferentially asymmetric divisions and formation of the ICM (Jedrusik et al., 2008). Asymmetric divisions are also facilitated by the expression of methylotransferase Carm1, which inhibits expression of Cdx2 (Parfitt and Zernicka-Goetz, 2010). Carm1 and Cdx2 levels correlate also with the expression of polarity markers such as aPKC or Par3: high expression of Cdx2 (low expression of Carm1) enhances apical accumulation of aPKC and Par3 (Jedrusik et al., 2008; Parfitt and Zernicka-Goetz, 2010). Although plane of cell division has a crucial meaning for the fate of the progeny of the dividing cell, the mechanism regulating the division orientation in mammalian embryos remains largely unknown.

## Results and discussion

### Position of the nucleus and the plane of cell division in 8-cell embryos

**Fig. 1:**
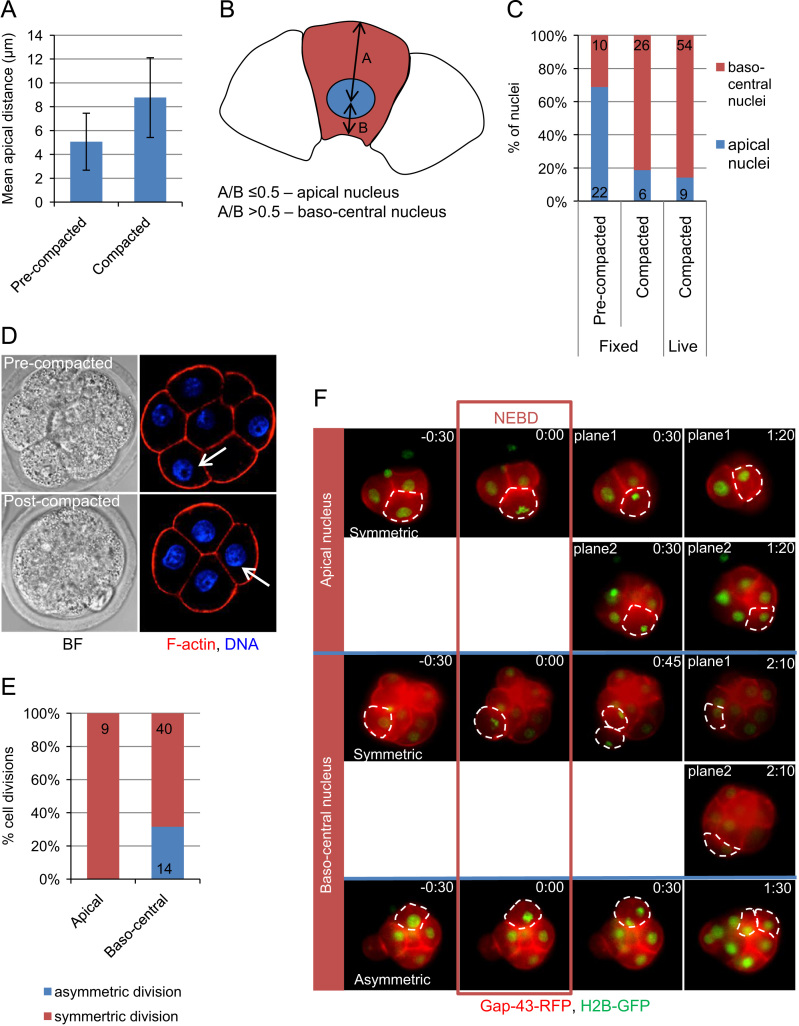
Translocation of nuclei at the 8-cell stage and the plane of cell division. (A) Mean distance between centre of the nucleus and apical membrane (so called apical distance) in pre- and post-compacted 8-cell embryos. The error bars represent standard deviations. (B) Scheme showing classification criteria for apical and baso-central nuclei. Distance between centre of the nucleus and apical–basal cortex was measured in embryos using ImageJ software. If the ratio between the apical and the basal distances was smaller than 0.5, the nucleus was classified as apical. Otherwise, it was classified as located baso-centrally. (C) Change in percentage of apical and baso-central nuclei in pre-compacted (fixed) and post-compacted (fixed or live) 8-cell embryos. Numbers in the graph reflect numbers of analysed nuclei. (D) Pre- and post-compacted 8-cell embryos. Arrows indicate an apical nucleus in the pre-compacted embryo and a baso-central nucleus in the post-compacted embryo. F-actin stained with phalloidin-TexasRed in red and DNA stained with Hoechst 33342 in blue. (E) Frequency of symmetric and asymmetric divisions in cells with apically and baso-centrally located nuclei. Numbers in the graph reflect numbers of analysed divisions. (F) Cells with nuclei located apically 30 min before the nuclear envelope break-down (NEBD) tend to divide symmetrically (the first two rows of images), whereas cells with baso-central nuclei divide either symmetrically (two central rows of the images) or asymmetrically (the bottom row of the images). In some cases images from two different planes from the same time-point are shown. Time in the images is shown in hours and minutes (h:min) with the timepoint 0:00 at NEBD. Analysed cells are outlined with a dashed white line. Gap43-RFP, a membrane marker in red and histone H2B-GFP in green.

In order to gain insight into the mechanism regulating the plane of cell division in the early mouse embryo, we first wished to determine the events preceding symmetric vs. asymmetric divisions at the 8-cell stage. One of the most striking reorganizations occurring at this developmental stage is relocation of nuclei. While at the early 8-cell stage, all nuclei are located apically towards the end of the cell cycle, the nuclei of some, but not all, cells become repositioned towards the baso-central part of the cell (Reeve and Kelly, 1983). The biological meaning behind this has remained however unknown. To address this, it was important to first confirm this observation and examine its dynamics and mechanism. To this end, we determined the distance between the nucleus and apical domain at early (pre-compacted) and late (compacted) 8-cell stage, 8 h apart. We found that the apical-to-nucleus distance increases during this period >1.7 fold (5.1±2.4 µm (±SD) vs. 8.8±3.3 µm (±SD), *p*<0.0001) reflecting translocation of nuclei from an apical to a baso-central position (Fig. 1A). As a result the proportion of cells with an apical localization of the nucleus decreases significantly as 8-cell embryo undergoes compaction (69% in pre-compacted embryo vs. 19% in compacted embryos; total of 32 nuclei from 8 pre-compacted and 32 nuclei from 8 compacted embryos analysed, Fig. 1B–D).

To ensure that this relocation does not reflect an artefact of fixation, we analysed the exact behaviour of the nuclei in time-lapse studies. To this end, we injected synthetic mRNA for Gap43-RFP, as a membrane marker, into one blastomere of a 2-cell embryos expressing histone H2B-GFP, as a marker of nuclei. We filmed their development from the mid-8-cell stage, using fluorescent wide-field or spinning-disc confocal microscopy collecting images every 10–15 min on 12–15 focal planes for each time point, as described previously (Morris et al., 2010). The analyses of these movies revealed that 30 min before nuclear envelope break-down (NEBD) at the 8–16-cell transition only 14% of nuclei (9/63, 21 embryos) were located apically (Fig. 1C). Embryos were heterogeneous with respect to the number of apical and baso-central nuclei prior to the division. In most cases, the apical nuclei constitute 0–50% of the analysed blastomeres in individual embryos, on average 16% (±29%, SD). Nuclei localized baso-centrally constitute 67–100% of the analysed nuclei, on average 84% (±29%, SD). Importantly, time-lapse studies allowed us to determine the division planes at the 8–16-cell stage and relate it with the position of the nucleus. This revealed that in all cases asymmetric divisions occur when nuclei become re-positioned baso-centrally (14/14), while symmetric divisions occur when the nucleus is positioned either apically (18%, 9/49) or baso-centrally (82%, 40/49), (Fig. 1E and F) suggesting that for a cell to divide asymmetrically, its nucleus moves basally.

### Spatiotemporal pattern of the nuclear movement

**Fig. 2:**
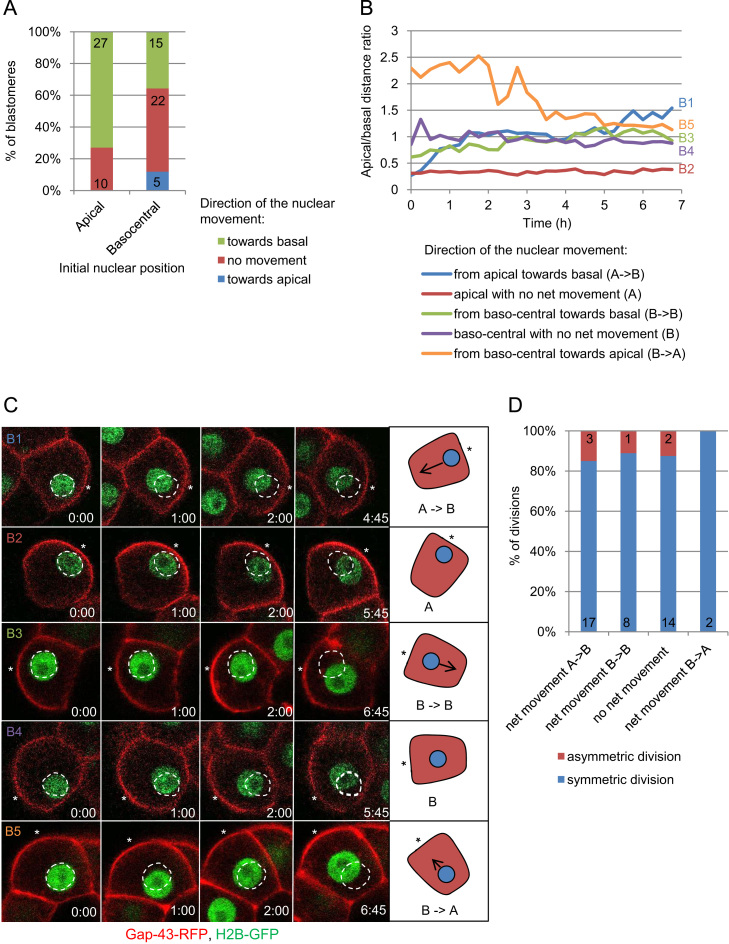
Dynamics of the nuclear movement in 8-cell embryo. (A) Direction of the net movement recorded for nuclei starting the 8-cell stage in the apical or baso-central position. (B) Dynamics of the nuclear movement throughout the 8-cell stage. Each line represents a ratio of apical to basal nuclear distances plotted over time for the representative blastomeres (B1–5) showing different patterns of the nuclear movement. Images of the same blastomeres are presented in (C). (C) Time-lapse images of the representative blastomeres (B1–5) illustrating the movement of their nuclei (the apical/basal distance ratio, a quantitative measure of the movement, is presented in (B)). Time in the images is shown in hours and minutes (h:min) with the timepoint 0:00 in the beginning of the analysis. Initial positions of the nuclei are marked by a dashed white circle. Asterisks mark the apical surfaces of the blastomeres. Gap43-RFP, a membrane marker in red and histone H2B-GFP in green. Cartoons on the right side of the panel represent schematically the type of movement that is captured in the respective time-lapse images. (D) Frequency of symmetric and asymmetric divisions occurring in the cells with baso-central nuclei displaying different types of the net movement throughout the 8-cell cycle.

In order to better understand the developmental significance of the nuclear translocation, we followed the dynamics of the nuclear movement throughout the 8-cell stage. To this end we injected 2-cell embryos with synthetic mRNA for Gap43-RFP, as a membrane marker. H2B-GFP, a nuclear marker, was either expressed endogenously by the embryos or introduced by mRNA injection at 2-cell stage. The embryos (*n*=32) were recorded from late 4-cell or pre-compacted 8-cell stages until the 8–16-cell transition. In agreement with our previous observations, the majority of nuclei in 8-cell blastomeres were initially localized apically. As the cell cycle progresses, most of them move basally (73%, 27/37), but some maintain an apical position (27%, 10/37). On the other hand, all nuclei that start the 8-cell stage in a baso-central position maintain it: they either show no net movement (52%, 22/42) or move basally (36%, 15/42), and only few (12%, 5/42), move apically, staying however in the baso-central regions of the cell (Fig. 2A–C). In all cases the nuclear translocation was gradual, and usually occurred in the first half of the cell cycle (Fig. 2B and C). Its precise timing varied between blastomeres, even in the same embryo.

As blastomeres with nuclei located baso-centrally prior to the NEBD may divide either asymmetrically or symmetrically, we examined whether plane of the division is determined by the pattern of the nuclear movement. As we show in Fig. 2D, nuclei that moved to the baso-central location from an initial apical position and nuclei that maintained their baso-central position throughout the 8-cell stage were able to divide either symmetrically or asymmetrically. Only 2 analysed cells had nuclei that retained the baso-central position regardless of the net apical movement they displayed. Interestingly, they divided symmetrically, but due to the small number it is difficult to draw any conclusion from this observation. Together, our analysis shows that although nuclei display different dynamics and direction of the movement, the exact pattern does not seem to be important for the plane of 8–16-cell division.

### Re-positioning of the nucleus is microtubule- and kinesin-dependent

**Fig. 3:**
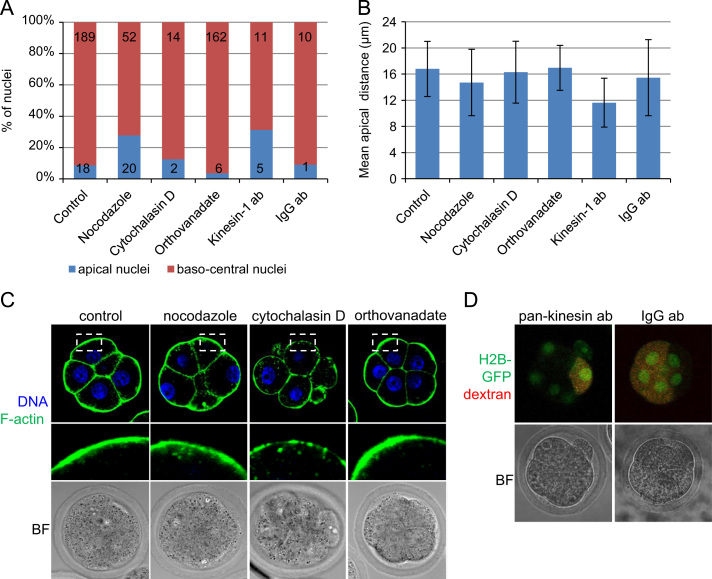
Role of the cytoskeleton in translocation of nuclei in 8-cell embryo. (A) Frequency of symmetric and asymmetric divisions with apically and baso-centrally located nuclei treated with nocodazole, cytochalasin D, sodium orthovanadate, injected with pan-kinesin antibody or control IgG antibody. Numbers in the graph reflect numbers of analysed divisions. (B) Mean distance between centre of the nucleus and apical membrane (so called apical distance) in late 8-cell embryos treated with nocodazole, cytochalasin D, sodium orthovanadate, injected with pan-kinesin antibody or control IgG antibody. The error bars represent standard deviations. (C) Localization of nuclei in late 8-cell embryos treated with nocodazole (to depolymerize microtubules), cytochalasin D (to depolymerize F-actin) and sodium orthovanadate (to inhibit dynein). F-actin stained with phalloidin-OregonGreen in green and DNA stained with Hoechst 33342 in blue. Apical regions outlined with a dashed line are showed in higher magnification. (D) Localization of nuclei in late 8-cell embryos injected with pan-kinesin antibody (to block kinesins) or control IgG antibody. Rhodamine-dextran, used as a lineage tracer, in red, histone H2B-GFP in green.

To determine the mechanism of nuclei translocation, we examined its dependence on cytoskeletal components. To depolymerize microtubules, we used nocodazole (5 µg/ml) as previously (Ajduk et al., 2011; Gong et al., 2010). We found that in nocodazole-treated embryos, 28% of nuclei (20/72, 11 embryos) maintained their apical position, whereas in control embryos only 9% of nuclei (18/207, 32 embryos) failed to leave the apical domain (*p*<0.0001) (Fig. 3A). Thus, upon nocodozale treatment the mean distance between the nucleus and the apical membrane was significantly lower in comparison to the control cells (14.7±5.1 µm (±SD) vs. 16.8±4.2 µm (±SD), *p<*0.001) (Fig. 3B and C). To depolymerize actin filaments, we used cytochalasin D (2 µg/ml) (Ajduk et al., 2011). We found that this treatment, although blocked the embryo compaction, had no effect upon the behaviour of nuclei: nuclei moved baso-centrally with a frequency similar to that observed in control, untreated embryos (88%, 14/16, 5 embryos vs. 91%, 189/207, 32 embryos, respectively) (Fig. 3A). There were also no significant differences between nuclear apical distances achieved in cytochalasin D-treated and control embryos (Fig. 3B and C).

Since these results suggest that translocation of the nuclei depends on microtubules, we investigated which motor proteins might be involved. Motor proteins responsible for the transport of organelles along microtubules form two families: the kinesin family, most members of which mediate transport towards the plus-end, and dyneins which support the minus-end transport (Hirokawa, 1998). Both types of motor proteins can associate with nuclear envelope either through the SUN–KASH complex (Crisp et al., 2006; McGee et al., 2006; Tapley and Starr, 2013) or through interactions with nuclear pore components (Cai et al., 2001; Padmakumar et al., 2005; Payne et al., 2003; Splinter et al., 2010) to permit movement of the nucleus along microtubules. To address whether dyneins might be involved in this process, we cultured 8-cell embryos in the presence of sodium orthovanadate (500 µM), a phosphatase inhibitor with a high level of selectivity for dynein over kinesins (Niclas et al., 1996). This treatment did not affect the mean distance between nuclei and apical membrane in comparison to control embryos. However, after orthovanadate treatment, 96% of nuclei moved baso-centrally (162/168), more (*p*<0.05) than in control group (Fig. 3A), suggesting that inhibition of dynein could facilitate the baso-central translocation of the nucleus. Indeed, in many polar cell types dynein localizes in the cortex and has been reported to impose a mechanical pulling force on microtubules attached to the nucleus, leading in turn to the nuclear dislocation (Dujardin and Vallee, 2002; Gonczy, 2008; Laan et al., 2012a,b; Neumuller and Knoblich, 2009).

To determine whether kinesin-dependent movement towards microtubule plus-end is required for the baso-centrally directed translocation of the nuclei, we injected early 8-cell blastomeres with pan-kinesin antibody mixed with rhodamine dextran, as a tracer, and checked the position of nuclei in post-compacted 8-cell embryos (*n*=11). The mean distance between nuclei and apical membrane in cells injected with pan-kinesin antibody was significantly shorter than in cells injected with control IgG antibody (11.6±3.7 µm (±SD) vs. 15.5±5.8 µm (±SD), *p*<0.05) (Fig. 3B and D). In addition, in 31% (5/16) of cells injected with kinesin antibody, nuclei failed to move towards the centre, whereas in cells injected with control IgG antibody only 9% of nuclei (1/11, 6 embryos) maintained the apical localization (Fig. 3A). This suggests that movement of nuclei towards baso-central part of the cell depends on kinesins and might be counteracted by dynein-driven movement towards the apical cortex.

### Cdx2, nuclear position and division plane in the 8-cell embryo

**Fig. 4:**
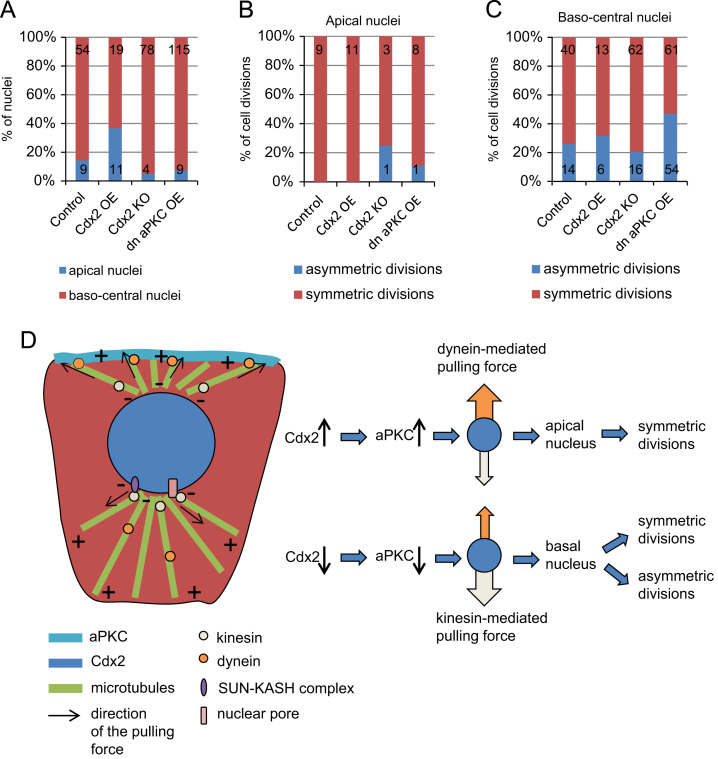
Relationship between Cdx2 and aPKC expression and localization of nuclei in 8-cell embryos and cell division plane. (A) Percentage of apical and baso-central nuclei in control embryos, embryos injected with exogenous Cdx2 or dn aPKC mRNAs (Cdx2 OE or dn aPKC OE, respectively) or Cdx2KO embryos at the 8-cell stage. Localization of the nuclei was assessed 30 min before NEBD. Numbers in the graph reflect numbers of analysed nuclei. (B) and (C) Frequency of symmetric and asymmetric divisions in cells with apical (B) or baso-central (C) nuclei from control, injected with exogenous Cdx2 or dn aPKC mRNAs (Cdx2 OE or dn aPKC OE, respectively) or from Cdx2KO embryos. Numbers in the graph reflect numbers of analysed divisions. (D) Working model of the interactions between Cdx2, aPKC, microtubules, dynein and kinesins and localization of nuclei and cell division orientation. Increase in Cdx2 expression leads to accumulation of aPKC in the apical site and in consequence reinforces dynein-mediated pulling force that facilitates apical localization of the nucleus. Cells with apical nuclei tend to divide symmetrically. Decrease in Cdx2 expression lowers accumulation of aPKC in the apical region and therefore tips the balance in favour of kinesin-mediated pulling force and baso-central localization of the nucleus. Cells with baso-central nuclei divide either symmetrically or asymmetrically.

The Cdx2 expression is initiated in a heterogeneous manner (Ralston and Rossant, 2008; Dietrich and Hiiragi, 2007) and this heterogeneity was shown to bias division orientation with cells expressing higher levels of Cdx2, dividing preferentially symmetrically rather than asymmetrically to contribute to the trophectoderm (Jedrusik et al., 2008). Therefore, we wished to test the hypothesis that the level of Cdx2 would correlate with the nuclear position. To this end, we first increased Cdx2 expression by injecting Cdx2 mRNA into one cell at the 2-cell stage as described by Jedrusik et al. (2008), together with Gap43-RFP mRNA (a membrane marker) into embryos expressing H2B-GFP (a nuclear marker). Embryos were cultured until the mid-8-cell stage and then filmed to reveal the division orientation. The analyses of the time-lapse movies revealed that in cells with a higher Cdx2 level, significantly more nuclei remained localized apically at the end of the cell cycle, in comparison to control embryos (37%, 11/30 nuclei, 9 embryos vs. 14%, 9/63 nuclei, 32 embryos) (Fig. 4A). Importantly, and in agreement with observations of control embryos, all cells with apically positioned nuclei divided symmetrically and all of the asymmetric divisions occurred in blastomeres with baso-central nuclei (Fig. 4B and C). This increased pool of cells with apically located nuclei accords with the tendency of cells overexpressing Cdx2 to divide symmetrically (Jedrusik et al., 2008).

To address whether the converse, depletion of Cdx2, would lead to an opposite effect, a decrease in number of apically located nuclei, we analysed the division planes in embryos of Cdx2KO^Zp3Cre^ females mated with heterozygous Cdx2^+/−^ males. Embryos from the above crosses were either devoid of only maternal Cdx2 or of both maternal and zygotic Cdx2, thus in both cases these embryos (referred to as Cdx2KO embryos) were expected to contain less Cdx2 at 8-cell stage than wild type embryos. To address whether Cdx2 depletion affects the division plane, one or both 2-cell blastomeres of Cdx2KO embryos were injected with H2B-RFP and Gap43-GFP mRNAs to visualize nuclei and cell membranes. In Cdx2KO embryos (*n*=31), significantly fewer nuclei stayed in an apical position in comparison to control embryos (5%, 4/82 vs. 14%, 9/63, *p*<0.05) (Fig. 4A). Of the total divisions recorded for the Cdx2KO, 19% were asymmetrical and of these all but one (94%, 16/17) occurred in cells in which nuclei moved towards basal region (Fig. 4B and C). Together, these results suggest that the level of Cdx2 expression can affect position of the nucleus and division symmetry.

### PKC activity, position of the nuclei and division plane in 8-cell embryo

Increased expression of Cdx2 is known to facilitate accumulation of aPKC in the apical region (Jedrusik et al., 2008). We therefore hypothesized that the aPKC accumulation may be a link between Cdx2 expression and localization of the nucleus. To test this hypothesis, we wished to determine the effect of the down-regulation of aPKC activity on the position of nuclei and symmetry of cell division. To this end, we injected one 2-cell stage blastomere with mRNAs of dominant negative form of aPKC (dn aPKC), H2B-RFP and Gap43-GFP and imaged embryos (*n*=48) during 8–16-cell transition. Only 7% (9/124) of nuclei in the injected clone of cells were localized apically and a great majority of them (89%, 8/9) divided symmetrically (Fig. 4A and B). Moreover, cells expressing dn aPKC divided asymmetrically twice more often than control embryos (44%, 55/124 vs. 22%, 14/63), as previously (Plusa et al., 2005). In agreement with our earlier results, the great majority of these asymmetric divisions (98%, 54/55) originated from cells with nuclei localized baso-centrally (Fig. 4C). It is plausible that aPKC localized in the apical region affects the pulling force exerted on microtubules linking the nucleus with the apical cortex. In *Caenorhabditis elegans* and *Drosophila* aPKC may cooperate with heterotrimeric G proteins to regulate microtubule motor protein dynein (Neumuller and Knoblich, 2009; Nguyen-Ngoc et al., 2007; Park and Rose, 2008; Srinivasan et al., 2003; Suzuki and Ohno, 2006). Interestingly, we find here that dynein is indeed involved in maintaining apical localization of the nuclei.

In conclusion, our results suggest that the extent of Cdx2 and aPKC expression, together with microtubule cytoskeleton, affect localization of nuclei in the 8-cell mouse embryo and, consequently, the plane of the cell division (Fig. 4D). In this model, the higher expression of Cdx2, the more intensely aPKC accumulates in the apical region which, in turn, facilitates pulling forces excreted by dyneins on microtubules attached to the nucleus to help sustain its apical localization. Cells with apically located nuclei divide almost always symmetrically. On the other hand, when Cdx2 is low, the apically directed pulling force is weakened and the nucleus is moved by kinesins along microtubules towards the baso-central region of a cell. Such cells with nuclei positioned baso-centrally maintain a flexibility regarding the division plane. Effectively all asymmetric divisions take place in cells with nuclei localized towards the basal region but these are still the minority of this group. Our results therefore suggest that apart from the position of the nucleus, other factors influence the cleavage plane. In agreement with this, the effect of misregulating the cell polarity network, for example through dn aPKC, has a greater effect upon the proportion of asymmetric divisions than it does on nuclear positioning. Thus, it is important to place the mechanism for nuclear positioning identified here into the context of other factors affecting spindle positioning, such as cell polarity itself, to understand how division is oriented in the mouse embryo.

## Materials and methods

### Animals

F1 (C57Bl6xCBA) and Cdx2 oocyte-specific Cre-mediated knock-out (Cdx2KO^Zp3Cre^) (Gao et al., 2009; Kaneda et al., 2009) mouse females and F1, Histone 2B-GFP (H2B-GFP) (Hadjantonakis and Papaioannou, 2004) and heterozygous Cdx2^+/−^ males (Chawengsaksophak et al., 1997) were used for the experiments. Animals were maintained in the Animal Facility of Gurdon Institute at 12:12 light cycle and provided with food and water ad libitum. Experiments were conducted in compliance with the University of Cambridge regulations.

### Embryo collection and culture

Females were superovulated with intraperitoneal injection of 7.5 IU of pregnant mare serum gonadotrophin (Intervet) and 48 h later of 7.5 IU of human chorionic gonadotrophin (Intervet). 2-Cell embryos were recovered 42 h later from oviducts into M2 medium and cultured in KSOM medium until 8-cell stage, as described before (Bischoff et al., 2008). In some experiments pre-compacted 8-cell embryos were moved to M2 medium and cultured for 8 h (until late G2-phase) with 5 µg/ml nocodazole, 2 µg/ml cytochalasin D, 500 µM sodium orthovanadate.

### Microinjections and live imaging of embryos

Constructs encoding Cdx2, dn aPKC, and Gap43 tagged with RFP or GFP were cloned into a pBluescript RN3P vector and mRNA was synthesized from T3 promotor using mMessage mMachine T3 kit (Ambion), as established previously (Zernicka-Goetz et al., 1997). Construct encoding Histone 2B tagged with RFP was cloned into pGEMHE vector and synthesized from T7 promotor using mMessage mMachine T7 kit (Ambion). mRNAs (0.05 μg/μl for Cdx2, 0.23 μg/μl for dn aPKC, 0.34 μg/μl for Gap43-RFP and Gap43-GFP, 0.05 μg/μl for H2B-RFP) were injected into one 2-cell embryo blastomere and injected embryos were cultured in KSOM until late 4- or 8-cell stage. In some experiments 8-cell embryos׳ cells were injected with either rabbit pan-kinesin antibody (Cytoskeleton, 250 µg/ml), or control rabbit IgG (250 µg/ml). In both cases rhodamine dextran was used as a cell lineage tracer. Embryos were transferred to M2 medium and imaged in 12–15 planes (5–7 µm apart) every 15 min for 12 h over the 8–16-cell transition. Imaging was performed on Leica scanning confocal microscope, Zeiss spinning-disc confocal microscope or Deltavision fluorescence microscope, equipped with 37.5 °C chambers.

### Immunostaining

Embryos were fixed in 4% PFA (30 min, RT), permeabilised with 0.2–0.5% Triton-X100 (30 min, RT) and blocked with 3% BSA. Cortical actin was stained with phalloidin labelled with TexasRed or OregonGreen (Invitrogen; 1:100, 30 min, RT or overnight, 4 °C) and DNA with Hoechst 33342 dye (Molecular Probes; 100 ng/µl in PBS, 30 min, RT or overnight, 4 °C).

### Statistical analysis

Statistical analyzes were performed either using Student׳s *t*-test, chi-squared test or exact Fisher test. In the analysis all blastomeres were treated individually, even when they originated from the same embryo.
